# Factors associated with high-risk human papillomavirus test utilization and infection: a population-based study of uninsured and underinsured women

**DOI:** 10.1186/s12905-018-0656-3

**Published:** 2018-10-03

**Authors:** Adana A. M. Llanos, Jennifer Tsui, David Rotter, Lindsey Toler, Antoinette M. Stroup

**Affiliations:** 1 Department of Epidemiology, Rutgers School of Public Health, 683 Hoes Lane West, Room 211, Piscataway, NJ 08854 USA; 2 Division of Population Science, Rutgers Cancer Institute of New Jersey, New Brunswick, NJ USA; 3 Department of Social and Behavioral Health Sciences, Rutgers School of Public Health, Piscataway, NJ USA; 4 New Jersey State Cancer Registry, New Jersey Department of Health, Trenton, NJ USA

**Keywords:** Cervical cancer screening, HPV, Co-testing, Minority populations, Medically underserved, Uninsured, Disparities

## Abstract

**Background:**

Current cervical cancer screening guidelines recommend a Pap test every 3 years for women age 21–65 years, or for women 30–65 years who want to lengthen the screening interval, a combination of Pap test and high-risk human papilloma virus testing (co-testing) every 5 years. Little population-based data are available on human papilloma virus test utilization and human papilloma virus infection rates. The objective of this study was to examine the patient-level, cervical cancer screening, and area-level factors associated with human papilloma virus testing and infection among a diverse sample of uninsured and underinsured women enrolled in the New Jersey Cancer Early Education and Detection (NJCEED) Program.

**Methods:**

We used data for a sample of 50,510 uninsured/underinsured women, age ≥ 29 years, who screened for cervical cancer through NJCEED between January 1, 2009 and December 31, 2015. Multivariable logistic regression models were used to estimate associations between ever having a human papilloma virus test or a positive test result, and individual- (age, race/ethnicity, birthplace) and area-level covariates (% below federal poverty level, % minority, % uninsured), and number of screening visits.

**Results:**

Only 26.6% (13,440) of the sample had at least one human papilloma virus test. Among women who underwent testing, 13.3% (1792) tested positive for human papilloma virus. Most women who were positive for human papilloma virus (99.4%) had their first test as a co-test. Human papilloma virus test utilization and infection were significantly associated with age, race/ethnicity, birthplace (country), and residential area-level poverty. Rates of human papilloma virus testing and infection also differed significantly across counties in the state of New Jersey.

**Conclusions:**

These findings suggest that despite access to no-cost cervical cancer screening for eligible women, human papilloma virus test utilization was relatively low among diverse, uninsured and underinsured women in New Jersey, and test utilization and infection were associated with individual-level and area-level factors.

## Background

Approximately 80 million people are currently infected and 14 million people are newly infected annually with human papillomavirus (HPV) in the United States (US) [1]. Of the over 150 types of HPV identified to date, 13 of them (HPV 16, 18, 31, 33, 35, 39, 45, 51, 52, 56, 58, 59, and 68) are recognized as human carcinogens and are considered high-risk (HR-HPV) types for cervical cancer [2]. Although all HR-HPVs are associated with increased risk of certain cancers, HPV 16 and 18 are considered the most carcinogenic, as persistent infection of these HR-HPVs are responsible for approximately 70% of all cervical cancers in women [3].

In 2004, HR-HPV DNA tests were approved by the US Food and Drug Administration (FDA) for use concurrently with cytology (co-testing) as a primary cervical cancer screening test for women age ≥ 30 years [4]. Then in 2012, published cervical cancer screening guidelines recommended screening for cervical cancer in women aged 21 to 65 years with cytology (Papanicolaou smear) every 3 years or, for women aged 30 to 65 years who want to lengthen the screening interval, screening with a combination of cytology and HPV testing every 5 years [5–7]. Most recently, in 2014, the FDA approved the first HPV DNA test for primary cervical cancer screening in women aged 25 years and older [8].

Little population-based data exist on the utilization of and barriers/facilitators to HPV DNA testing in the US, particularly for minority and medically underserved women with a higher risk of developing cervical cancer. The limited population-based studies on HPV co-test utilization have generally demonstrated low utilization across various population subgroups (approximately ≤20% testing rates) [9–12]; however, more recent data suggest increased HPV co-test utilization by mid-2013 through 2014 in some populations (≥44% to as high as 78%) [13, 14]. Recent analysis of the National Breast and Cervical Cancer Early Detection Program (NBCCEDP) have suggested a variety of provider-level factors (e.g., provider characteristics, practices, beliefs, etc.) are associated with cervical cancer screening overall and HPV co-test utilization in the NBCCEDP [9, 15–18]. However, few patient- and area-level characteristics or disparities in HR-HPV DNA test utilization across population subgroups were examined.

Although rates of cervical cancer incidence in New Jersey has been declining dramatically from 1990 (15.0 per 100,000) to 2014 (7.5 per 100,000) at approximately 2.7% per year (95% CI -2.9, -2.4) [14], substantial variation exists in cervical cancer incidence within the state by geographic and population subgroups. For example, Hispanic women in New Jersey and women of all race/ethnicities residing in at least four counties within the state have cervical cancer incidence rates that are significantly higher than the US average [14]. The objective of this study was to examine the patient-, clinical-, and area-level factors associated with HR-HPV DNA testing and HR-HPV infection among a diverse sample of uninsured and underinsured women enrolled in the New Jersey Cancer Early Education and Detection (NJCEED) Program, which is part of the NBCCEDP.

## Methods

### Study sample

**Fig. 1:**
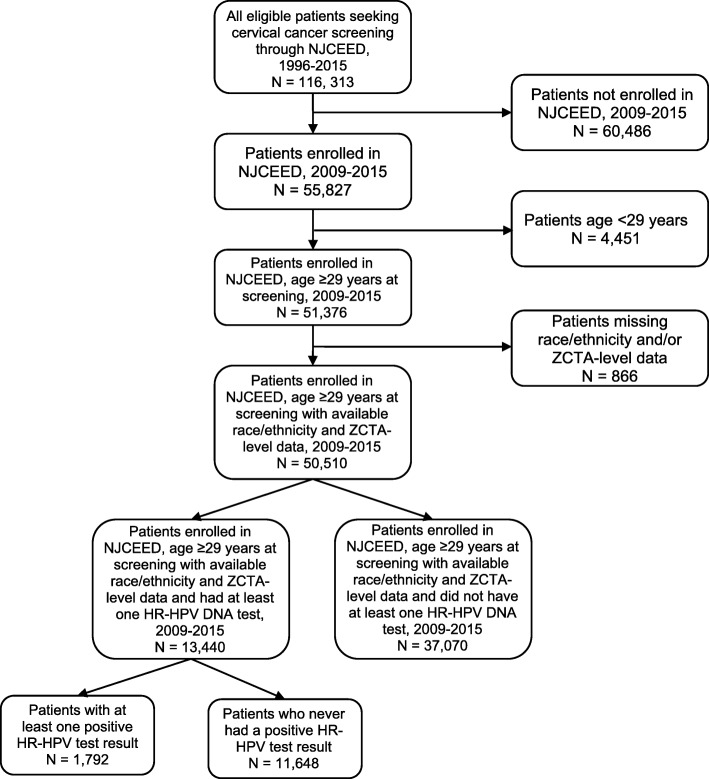
Flow diagram describing the selection of the analytic cohort

The NJCEED Program is housed within the New Jersey Department of Health (NJDOH) and is funded primarily by the NJDOH and the Centers for Disease Control and Prevention (CDC) to provide screening, diagnostic, and treatment services to eligible individuals for breast, cervical, prostate and colorectal cancers. Eligibility for receipt of NJCEED program services, include low-income (at or below 250% of the Federal Poverty Level [FPL]) and lack of adequate health insurance (uninsured or underinsured). NJCEED has been providing breast and cervical cancer outreach, education, early detection, screening and follow-up to eligible individuals in New Jersey since 1993 with initial funding through the CDC’s Preventive Health and Health Services Block Grant. Statewide coverage, with services being provided in all 21 counties, began on September 1, 1997. Currently, NJCEED program services are provided through 21 contracted lead agencies, with at least one lead agency in each county. The lead agencies work directly with providers within their county to provide services to eligible program participants. Between January 1, 2000 and December 31, 2015, a total of 116,313 unique and eligible women, age 21 to 64 years, received cervical cancer screening services through NJCEED. Among these, 55,827 were enrolled during the study period of interest (January 1, 2009 through December 31, 2015). We further restricted our study sample to women age ≥ 29 years, who had available data on race/ethnicity and residential zip code (Fig. 1).

### Data collection

A limited data set was extracted and compiled by NJCEED from the Program’s Cancer Screening and Tracking System (CaST), which includes data on invasive cancer cases ascertained through annual data linkages with the New Jersey State Cancer Registry (NJSCR). CaST allows the user to track patients through the length of the study and collect information on screening and diagnostic procedures done for breast and cervical cancer (NBCCDEP) and colorectal cancer (CRCCP). Individual-level data for this study were obtained from the Minimum Data Elements (MDEs) of the CaST based on inclusion criteria described above. All identifiers were removed prior to providing the limited data set to study investigators for analysis. Use of the data and all related study activities were approved by the New Jersey Department of Health, Rutgers Biomedical Health Sciences Institutional Review Board, and the Scientific Review Board of Rutgers Cancer Institute of New Jersey.

### Patient-level and cervical cancer screening measures

Age at enrollment was defined as the age at the earliest cervical cancer screening visit during the study period (2009–2015). Race/ethnicity and place of birth were based on patient self-report and stored in CaST. A total of 166 unique countries of birth were reported among participants, which were collapsed into 7 categories: (1) USA, (2) Central and South America, (3) Caribbean, (4) Africa, (5) Asia and the Middle East, (6) Europe, Russia, Australia and Oceania, and (7) Other Countries. In terms of cervical cancer screening variables, number of cervical cancer screening visits was defined as the total number of unique visits to NJCEED providers for any cervical cancer screening procedures at any time during the study period (e.g., gynecologic consultation, Pap test, pelvic exam, HPV test); and number of HR-HPV DNA tests was defined as the total number of unique visits where an HPV test was performed during the study period. HR-HPV DNA test results were only available as positive result or negative result. Specific HR-HPV DNA types (e.g., 16, 18, etc.) for positive results were not available. Age at first Pap test and HPV test were defined as study participant’s age at the earliest Pap test or HPV test, respectively, reported during the study period.

### Area-level measures

Residential zip code tabulation area (ZCTA) for each study participant was linked to the 2010–2014 American Community Survey (ACS) 5-year ZCTA-level estimates collected by the US Census Bureau. The following ACS estimates were obtained to derive area-level sociodemographic factors: (1) poverty status in the past 12 months; (2) race/ethnicity; (3) health insurance coverage type; and (4) language spoken at home. Proportions of participants falling into quintiles (1 = low, 5 = high) of each of the above sociodemographic factors (based on ZCTA population estimate distributions within the NJCEED population in our study cohort) were estimated. The proportion of minority residents per ZCTA was calculated as the difference of 1 minus the proportion of non-Hispanic White (NHW) residents. Age-adjusted cervical cancer incidence and mortality rates by New Jersey county were obtained from the National Cancer Institute (NCI) State Cancer Profiles [14], which summarizes data from the latest SEER submission (December 2016).

### Statistical analysis

Our primary outcomes of interest are having at least one HR-HPV DNA test and having ever received a positive HR-HPV DNA test. Descriptive statistics were used to summarize the individual sociodemographic and screening characteristics. Chi-square tests were used to compare these variables by HR-HPV DNA test receipt and receipt of a positive HR-HPV DNA test result. We conducted bivariate logistic regression models to examine the relationship between individual-level covariates (age at enrollment, race/ethnicity, country of birth), number of screening visits, and area-level measures with our primary outcomes. Full models were based on significant relationships in the unadjusted models. Tests for multicollinearity were also conducted, with negative results. We ran multivariate models adjusting for year, which was significantly associated with HR-HPV DNA testing rates but not HR-HPV positivity. Upon inclusion of year in the full model, the associations remained mostly consistent. Study participants with missing demographic or zip codes were excluded from analysis. We estimated odds ratios (OR) and 95% confidence intervals (CI) using robust standard errors and defined significance as *P* < 0.05 level. County level rates of HR-HPV DNA testing, positive results for HR-HPV DNA test, and invasive cervical cancer incidence and mortality were also examined. Data were analyzed using SAS v9.4 (SAS Institute, Cary, NC) and STATA v14 (StataCorp, College Station, TX).

## Results

Among the 50,510 women included in the analysis, 26.6% (13,440) had at least one HR-HPV DNA test through NJCEED over the 6-year study period from 2009 to 2015. Among those who ever had a HR-HPV DNA test, 13.3% (1792) had received a positive test result. Characteristics of the analytic cohort overall, as well as among those ever tested for HR-HPV and among those who ever received a positive HR-HPV test result are shown in Table 1. Overall, a large majority of the study sample was ≥40 years at enrollment in NJCEED (40–49 years, 39.4%; ≥50 years, 44.8%), of Hispanic ethnicity (53.4%), and foreign-born (70.9%).

**Table 1:** Characteristics of women ≥29 years who sought cervical cancer screening services through NJCEED, overall and among those who ever had a high-risk human papilloma virus (HR-HPV) DNA test and those who ever received a positive HR-HPV test result, 2009–2015

*Patient-level characteristics*	Total, *N* = 50,510	Ever had a HR-HPV DNA test, *n* = 13,440*		Ever received a positive HR-HPVtest result, *n* = 1792**	
	n (%)	n (%)	*P* ^*a*^	n (%)	*P* ^*b*^
Sociodemographics					
Age at enrollment into NJCEED (years)			**< 0.001**		**< 0.001**
29–39	7987 (15.8)	2457 (18.3)		532 (29.7)	
40–49	19,909 (39.4)	5663 (42.1)		689 (38.4)	
≥ 50	22,614 (44.8)	5320 (39.6)		571 (31.9)	
Race/ethnicity			**< 0.001**		**< 0.001**
Non-Hispanic White	12,420 (24.6)	2706 (20.1)		443 (24.7)	
Non-Hispanic Black	7716 (15.3)	1993 (14.8)		247 (13.8)	
Asian/Pacific Islander	3351 (6.6)	1060 (7.9)		98 (5.5)	
Hispanic	27,023 (53.5)	7681 (57.2)		1004 (56.0)	
Place of birth			**< 0.001**		**< 0.001**
USA	14,695 (29.1)	3468 (25.8)		525 (29.3)	
Central and South America	20,437 (40.5)	5575 (41.5)		754 (42.1)	
Caribbean	7266 (14.4)	2007 (14.9)		261 (14.6)	
Africa	952 (1.9)	243 (1.8)		19 (1.1)	
Asia and the Middle East	3283 (6.5)	1075 (8.0)		95 (5.3)	
Europe, Russia and Australia and Oceania	1549 (3.1)	546 (4.1)		61 (3.4)	
Other countries^c^	2328 (4.6)	526 (3.9)		77 (4.3)	
Cervical cancer screening characteristics					
Number of cervical screening visits			**< 0.001**		**< 0.001**
1	29,872 (59.1)	7399 (55.1)		842 (47.0)	
2	8760 (17.3)	2488 (18.5)		356 (19.9)	
≥ 3	11,878 (23.5)	3553 (26.4)		594 (33.1)	
Number of Pap tests			**< 0.001**		
0	1571 (3.1)	10 (0.1)		4 (0.2)	
1	35,670 (70.6)	8488 (63.2)		967 (54.0)	
2	8347 (16.5)	2717 (20.2)		379 (21.1)	
≥ 3	4922 (9.7)	2230 (16.6)		442 (24.7)	
Age at first Pap test (years)			**< 0.001**		
29–39	7778 (15.9)	2450 (18.2)		529 (29.6)	
40–49	19,291 (39.4)	5658 (42.1)		688 (38.5)	
≥ 50	21,870 (44.7)	5322 (39.6)		571 (31.9)	
Number of HPV tests			**< 0.001**		**< 0.001**
0	37,070 (73.4)	0 (0.0)		0 (0.0)	
1	11,810 (23.4)	11,810 (87.9)		1391 (77.6)	
2	1386 (2.7)	1386 (10.3)		306 (17.1)	
≥ 3	244 (0.5)	244 (1.8)		95 (5.3)	
Age at first HPV test (years)			–		**< 0.001**
29–39		2307 (17.2)		514 (28.7)	
40–49		5461 (40.6)		673 (37.6)	
≥ 50		5672 (42.2)		605 (33.8)	

*Abbreviations: FPL* federal poverty level, *HR-HPV* high-risk human papillomavirus, *NJCEED* New Jersey Cancer Early Education and Detection, *USA* United States of America, *ZCTA* ZIP Code Tabulation Area. Bolded values represent statistically significant associations (*P* <0.05)

*Of the 50,510 NJCEED patients ≥29 years included in the analytic sample, 13,440 (26.6%) had at least one HR-HPV DNA test

**Of the 13,440 NJCEED patients ≥29 years included in the analytic sample, 1792 (13.3%) had at least one positive HR-HPV DNA test result

^a^*P*-values were calculated using chi-square tests comparing women who ever had a HR-HPV DNA test to those who did not

^b^*P*-values were calculated using chi-square tests comparing women who ever had a positive HR-HPV DNA test result to those who did not

^c^Includes women born in Canada as well as those reporting other or unspecified countries outside the USA

In terms of cervical cancer screening, most women had only one screening visit (59.1%) and had only one Pap test (70.6%) over 6 years. Additionally, they had their first NJCEED Pap test an older age (40–49 years, 39.4%, ≥50 years, 44.7%). In terms of HR-HPV testing, 73.4% of the sample were never tested. HR-HPV DNA testing rates among women age ≥ 29 years increased from 7.4% in 2009 to 35.4% in 2015 (*P* < 0.0001). Compared to women who were never tested for HR-HPV test, those who were tested were more likely to be younger at enrollment into NJCEED, younger at their first Pap and HR-HPV tests, to have more cervical cancer screening visits, to have more Pap tests, and were less likely to be NHW and US-born (all *P*-values < 0.001). Almost all women who had a HR-HPV DNA test through NJCEED had their first test as a co-test with Pap test (99.8%) and the first test result was positive among 12.7% of these women. Like women who had a HR-HPV test, participants who tested positive for HR-HPV were also more likely to be younger at enrollment in NJCEED, younger at their first Pap and HR-HPV DNA tests, to have more cervical cancer screening visits, and to have more Pap tests compared to those who never tested positive for HR-HPV (all *P*-values < 0.001). However, unlike the women who were tested, HR-HPV positive women were more likely to be NHW and US-born. Almost all women who had ever received a positive HR-HPV test results (99.4%) had their first test as a co-test with a Pap test and for 95.5% of them their first HR-HPV test was positive.

In multivariable-adjusted models, we observed significant associations between age, race/ethnicity, country of birth, cervical cancer screening behaviors, and area-level poverty and having at least one HR-HPV DNA test (Table 2). Women who had their first cervical cancer screening visit at an older age (40–49 years, OR 0.83, 95% CI: 0.78–0.88; ≥50 years, OR 0.63, 95% CI: 0.59–0.67), were born in Central and South American countries (OR 0.86, 95% CI: 0.80–0.92) or the Caribbean (OR 0.91, 95% CI: 0.84–0.98), and resided in a ZCTA with lower proportions of residents below the FPL (Quintiles 1–4), had reduced odds of being tested for HR-HPV. Conversely, racial/ethnic minorities (NHB, OR 1.38, 95% CI: 1.28–1.49; Asian/Pacific Islander [API], OR 1.23, 95% CI: 1.03–1.46; Hispanic, OR 1.54, 95% CI: 1.43–1.66), women born in Asian and the Middle Eastern countries (OR 1.61, 95% CI: 1.35–1.91) or Europe, Russia, Australia or Oceania (OR 2.18, 95% CI: 1.94–2.45), women who had been screened for cervical cancer more than once (2 times, OR 1.60, 95% CI: 1.52–1.69; ≥3 times, OR 2.77, 95% CI: 2.61–2.94), and resided in a ZCTA with a high proportion of residents living below the FPL (Quintile 5, OR 1.50, 95% CI: 1.41–1.61) had increased odds of being tested for HR-HPV.

**Table 2:** Logistic regression analysis of factors associated with *having at least one HR-HPV DNA test* among women who sought cervical care through NJCEED, 2009–2015

	Proportion that ever had a HR-HPV DNA test (%)	Unadjusted OR (95% CI)	Multivariable-adjusted OR (95% CI)
Age at enrollment into NJCEED (years)			
29–39	30.8	1.00 (ref)	1.00 (ref)
40–49	28.5	**0.89 (0.84–0.95)**	**0.83 (0.78–0.88)**
≥ 50	23.5	**0.69 (0.65–0.73)**	**0.63 (0.59–0.67)**
Race/ethnicity			
Non-Hispanic White	21.8	1.00 (ref)	1.00 (ref)
Non-Hispanic Black	25.8	**1.25 (1.17–1.33)**	**1.38 (1.28–1.49)**
Asian/Pacific Islander	31.6	**1.66 (1.52–1.80)**	**1.23 (1.03–1.46)**
Hispanic	28.4	**1.42 (1.35–1.50)**	**1.54 (1.43–1.66)**
Place of birth			
USA	23.6	1.00 (ref)	1.00 (ref)
Central and South America	27.3	**1.21 (1.15–1.27)**	**0.86 (0.80–0.92)**
Caribbean	27.6	**1.23 (1.16–1.31)**	**0.91 (0.84–0.98)**
Africa	25.5	1.11 (0.95–1.31)	1.00 (0.85–1.17)
Asia and the Middle East	32.7	**1.57 (1.45–1.71)**	**1.61 (1.35–1.91)**
Europe, Russia and Australia and Oceania	35.3	**1.76 (1.58–1.97)**	**2.18 (1.94–2.45)**
Other countries^a^	22.7	0.95 (0.85–1.05)	0.95 (0.85–1.06)
Number of cervical screening visits			
1	22.7	1.00 (ref)	1.00 (ref)
2	31.5	**1.56 (1.48–1.64)**	**1.60 (1.52–1.69)**
≥ 3	43.1	**2.58 (2.43–2.73)**	**2.77 (2.61–2.94)**
Proportion of residents in ZCTA who are below the FPL			
Quintile 1 (0.0–6.9%)	27.2	1.00 (ref)	1.00 (ref)
Quintile 2 (7.0–10.8%)	23.3	**0.79 (0.74–0.85)**	**0.79 (0.74–0.85)**
Quintile 3 (10.9–18.5%)	23.7	**0.81 (0.76–0.87)**	**0.81 (0.76–0.87)**
Quintile 4 (18.7–23.7%)	21.9	**0.73 (0.69–0.78)**	**0.77 (0.72–0.82)**
Quintile 5 (24.4–100.0%)	35.9	**1.47 (1.38–1.56)**	**1.50 (1.41–1.61)**
Proportion of minority residents in ZCTA			
Quintile 1 (0.0–26.6%)	20.0	1.00 (ref)	
Quintile 2 (26.7–49.0%)	24.2	**1.27 (1.19–1.36)**	
Quintile 3 (50.4–76.4%)	34.0	**2.06 (1.93–2.19)**	
Quintile 4 (76.5–87.2%)	21.4	**1.09 (1.01–1.16)**	
Quintile 5 (87.8–98.8%)	32.9	**1.96 (1.84–2.09)**	
Proportion of uninsured residents in ZCTA			
Quintile 1 (0.0–13.0%)	24.4	1.00 (ref)	
Quintile 2 (13.1–20.3%)	22.8	**0.91 (0.86–0.98)**	
Quintile 3 (20.4–28.8%)	29.4	**1.29 (1.21–1.37)**	
Quintile 4 (29.2–37.9%)	26.3	**1.11 (1.04–1.18)**	
Quintile 5 (38.1–100.0%)	30.3	**1.35 (1.27–1.43)**	
Proportion of residents in ZCTA who don’t speak English very well			
Quintile 1 (0.0–5.7%)	19.0	1.00 (ref)	
Quintile 2 (5.8–11.3%)	24.4	**1.38 (1.29–1.47)**	
Quintile 3 (11.4–19.1%)	29.6	**1.79 (1.68–1.91)**	
Quintile 4 (19.2–34.3%)	35.7	**2.36 (2.21–2.52)**	
Quintile 5 (34.6–51.6%)	24.8	**1.41 (1.31–1.50)**	

Multivariable-adjusted model was adjusted for all variables listed. Area-level measures are based on the distributions of the NJCEED study sample. We also tested the effect of including year (which was significantly associated with HR-HPV DNA testing rates, and the observed associations remained consistent, except the association between age and HR-HPV DNA testing, which showed that when year is included in the model, there was a stronger inverse association for the 40–49 years age group (OR 0.46, 95% CI: 0.40–0.52). Bolded values represent statistically significant associations (*P* <0.05)

In multivariable-adjusted models, we also observed significant associations between age, race/ethnicity, country of birth, cervical cancer screening patterns, and area-level poverty and the odds of testing positive for HR-HPV (Table 3). Women who enrolled in NJCEED at an older age (40–49 years, OR 0.46, 95% CI: 0.40–0.52; ≥50 years, OR 0.40, 95% CI: 0.35–0.46), were NHB (OR 0.75, 95% CI: 0.62–0.90) or Hispanic (OR 0.66, 0.55–0.79), and born in Africa (OR 0.54, 95% CI: 0.33–0.89), Asia or the Middle East (OR 0.53, 95% CI: 0.33–0.86), or Europe, Russia, Australia or Oceania (OR 0.63, 95% CI: 0.47–0.84) had decreased odds of testing positive for HR-HPV. Women who were more frequently screened for cervical cancer (2 times, OR 1.31, 95% CI: 1.15–1.49; ≥3 times, OR 1.86, 95% CI: 1.64–2.11) and resided in a ZCTA with relatively low proportions of residents below the FPL (Quintiles 1–2) had increased odds of testing positive for HR-HPV.

**Table 3:** Logistic regression analysis of factors associated with ever *receiving a positive HR-HPV DNA test result* among women who had at least one HR-HPV DNA test through NJCEED, 2009–2015

	Proportion that ever received a positive HR-HPV test result (%)	Unadjusted OR (95% CI)	Multivariable-adjusted OR (95% CI)
Age at enrollment into NJCEED (years)			
29–39	21.7	1.00 (ref)	1.00 (ref)
40–49	12.2	**0.50 (0.44–0.57)**	**0.46 (0.40–0.52)**
≥ 50	10.7	**0.44 (0.38–0.50)**	**0.40 (0.35–0.46)**
Race/ethnicity			
Non-Hispanic White	16.4	1.00 (ref)	1.00 (ref)
Non-Hispanic Black	12.4	**0.72 (0.61–0.85)**	**0.75 (0.62–0.90)**
Asian/Pacific Islander	9.2	**0.52 (0.41–0.66)**	0.94 (0.58–1.51)
Hispanic	13.1	**0.77 (0.68–0.87)**	**0.66 (0.55–0.79)**
Country/region of birth			
USA	15.1	1.00 (ref)	1.00 (ref)
Central and South America	13.5	**0.88 (0.78–0.99)**	0.96 (0.81–1.15)
Caribbean	13.0	**0.84 (0.71–0.98)**	1.07 (0.88–1.29)
Africa	7.8	**0.48 (0.29–0.77)**	**0.54 (0.33–0.89)**
Asia and the Middle East	8.8	**0.54 (0.43–0.68)**	**0.53 (0.33–0.86)**
Europe, Russia and Australia and Oceania	11.2	**0.71 (0.53–0.93)**	**0.63 (0.47–0.84)**
Other countries^a^	14.6	0.96 (0.74–1.25)	1.01 (0.77–1.32)
Number of cervical screening visits			
1	11.5	1.00 (ref)	1.00 (ref)
2	14.0	**1.26 (1.11–1.43)**	**1.31 (1.15–1.49)**
≥ 3	18.5	**1.75 (1.55–1.98)**	**1.86 (1.64–2.11)**
Proportion of residents in ZCTA who are below the FPL			
Quintile 1 (0.0–6.9%)	11.7	1.00 (ref)	1.00 (ref)
Quintile 2 (7.0–10.8%)	14.6	**1.29 (1.10–1.52)**	**1.23 (1.04–1.45)**
Quintile 3 (10.9–18.5%)	16.1	**1.45 (1.23–1.70)**	**1.46 (1.24–1.72)**
Quintile 4 (18.7–23.7%)	12.6	1.09 (0.92–1.30)	1.15 (0.96–1.38)
Quintile 5 (24.4–100.0%)	12.5	1.08 (0.93–1.26)	1.12 (0.95–1.32)
Proportion of minority residents in ZCTA			
Quintile 1 (0.0–26.6%)	15.2	1.00 (ref)	
Quintile 2 (26.7–49.0%)	16.2	1.07 (0.91–1.26)	
Quintile 3 (50.4–76.4%)	11.0	**0.69 (0.59–0.81)**	
Quintile 4 (76.5–87.2%)	14.6	0.95 (0.80–1.13)	
Quintile 5 (87.8–98.8%)	11.8	**0.75 (0.64–0.87)**	
Proportion of uninsured residents in ZCTA			
Quintile 1 (0.0–13.0%)	13.0	1.00 (ref)	
Quintile 2 (13.1–20.3%)	15.7	**1.24 (1.05–1.46)**	
Quintile 3 (20.4–28.8%)	12.8	0.97 (0.83–1.14)	
Quintile 4 (29.2–37.9%)	13.2	1.01 (0.86–1.19)	
Quintile 5 (38.1–100.0%)	12.5	0.95 (0.81–1.12)	
Proportion of residents in ZCTA who don’t speak English very well			
Quintile 1 (0.0–5.7%)	16.5	1.00 (ref)	
Quintile 2 (5.8–11.3%)	14.5	0.86 (0.73–1.02)	
Quintile 3 (11.4–19.1%)	11.5	**0.66 (0.56–0.77)**	
Quintile 4 (19.2–34.3%)	12.3	**0.71 (0.61–0.83)**	
Quintile 5 (34.6–51.6%)	13.5	**0.79 (0.67–0.93)**	

Multivariable-adjusted model was adjusted for all variables listed. Area-level measures are based on the distributions of the NJCEED study sample. We also tested the effect of including year (which was significantly associated with HR-HPV DNA testing rates but not HR-HPV infection), and the observed associations remained consistent. Bolded values represent statistically significant associations (*P* <0.05)

**Fig. 2:**
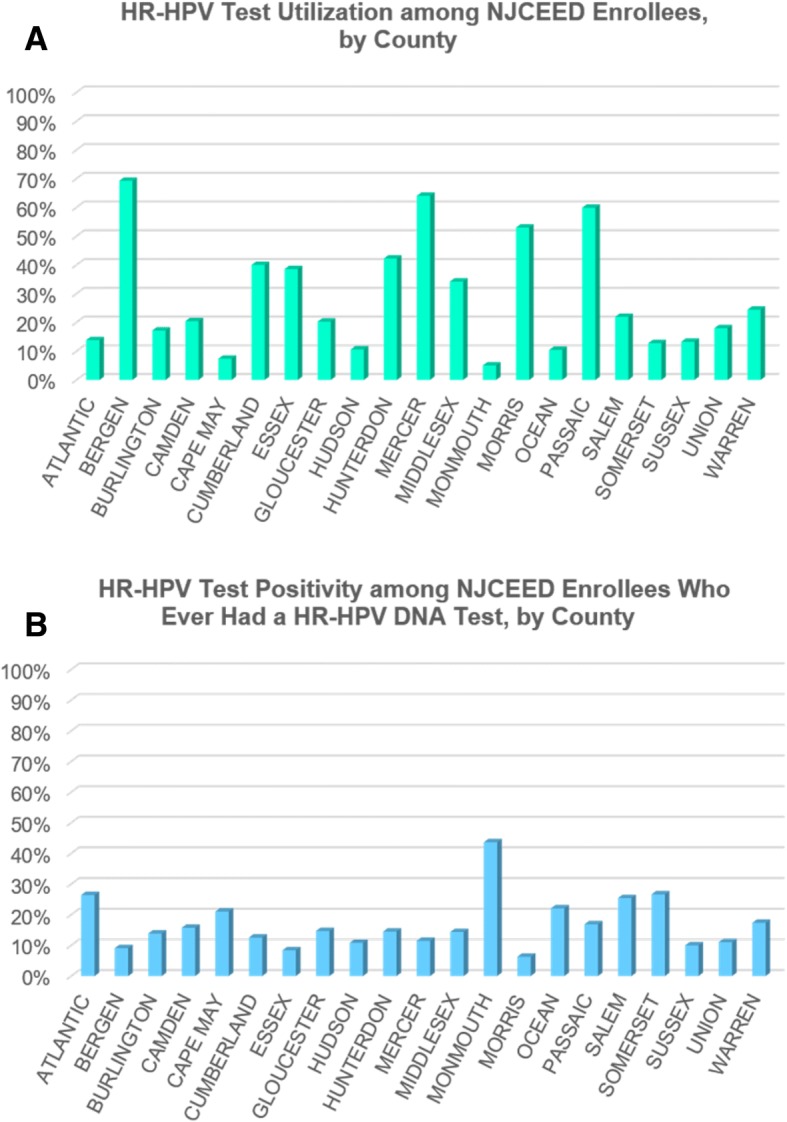
Bar graphs showing the distributions of high-risk human papillomavirus (HR-HPV) DNA test utilization (**a**) and receipt of at least one HR-HPV DNA positive test result (**b**) among women ≥29 years who sought cervical care through the New Jersey Cancer Education and Early Detection (NJCEED) Program, by New Jersey county of residence, 2009–2015. NOTE: The overall rate of HR-HPV testing among NJCEED participants was 26.6% (13,440 of the 50,510 unique patients had at least one HR-HPV DNA test) and the overall rate of having received at least on positive HR-HPV test result was 13.3% (1792 of the 13,440 patients who ever had a HR-HPV DNA test had at least one positive test result)

**Fig. 3:**
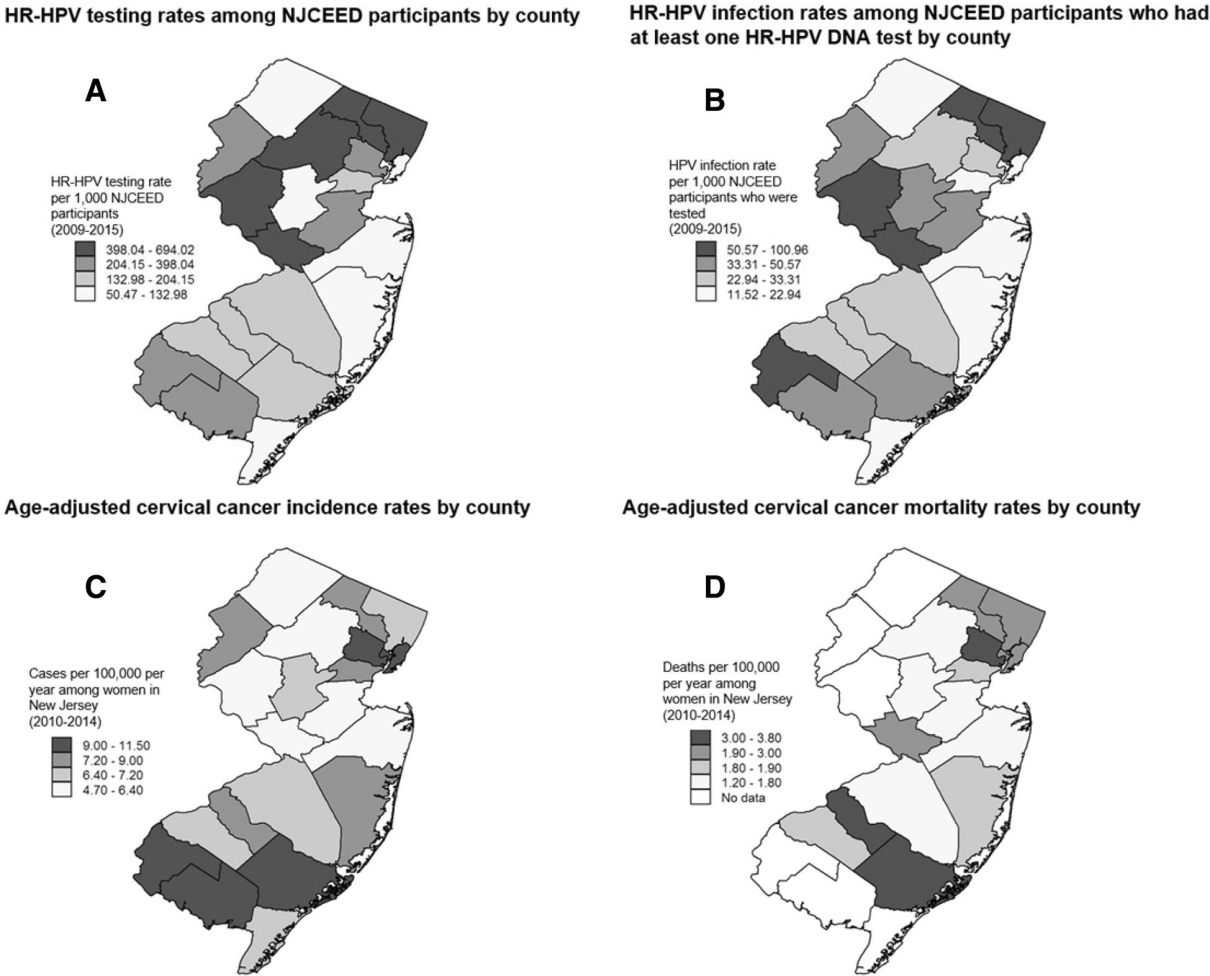
Maps showing high-risk human papillomavirus (HR-HPV) DNA testing rates among NJCEED participants during the study period (2009–2015), by county (**a**); HR-HPV infection among NJCEED participants who received at least one HR-HPV DNA test during the study period (2009–2015), by county (**b**); Age-adjusted cervical cancer incidence rates per 100,000 per year among all women in New Jersey (2010–2014), by county (**c**); and Age-adjusted cervical cancer mortality rates per 100,000 per year among all women in New Jersey (2010–2014), by county (**d**). NOTE: The rates of HR-HPV testing and infection are only among NJCEED participants (Fig. 3a and b), whereas the incidence and mortality rates of invasive cervical cancer are among all women in New Jersey (Fig. 3c and d). The maps depicted here are our own and were created using data from among the 50,510 NJCEED-enrolled women included in the analysis (Fig. 3a and b) and New Jersey State cervical cancer incidence and mortality data retrieved from www.statecancerprofiles.cancer.gov (Fig. 3c and d)

In our analysis of HR-HPV DNA testing and HR-HPV infection by New Jersey county of residence, we found that the rates of HR-HPV DNA testing among residents of eight counties were significantly higher than the 26.6% overall rate of testing in the study sample (Bergen, 69.4%; Mercer, 64.2%; Passaic, 60.0%; Morris, 53.0%; Hunterdon, 41.8%; Cumberland, 40.2%; Essex, 38.8%; and Middlesex, 34.2%; all *P*-values < 0.0001) (Fig. 2). In terms of HR-HPV infection, five of the counties with testing rates higher than the sample average had lower than the 13.3% overall average for HR-HPV infection (Bergen, 9.2%, *P* < 0.0001; Mercer, 11.7%, *P* = 0.002; Morris, 6.2%, P < 0.0001; Cumberland, 12.7%, *P* = 0.007, and Essex, 8.4%, P < 0.0001), while several other counties had HR-HPV infection rates that were 2- to 3-fold higher than the sample average (Atlantic, 26.3%; Somerset, 26.7%; and Monmouth, 44.2%; all P-values < 0.0001). Figure 3 shows HR-HPV testing and infection rates (per 1000 NJCEED participants) and age-adjusted cervical cancer incidence and mortality rates from 2010 to 2014 (per 100,000 New Jersey residents, based on NCI State Cancer Profiles data [14]). This figure shows that 4 of the 5 New Jersey counties with the highest rates of HPV testing (398.04–694.02 per 1000) also have the highest rates of HPV positivity (50.57–100.96 per 1000) (Bergen, Passaic, Hunterdon, and Mercer). Some counties with low to moderate rates of HPV testing among NJCEED participants are among the counties (Atlantic, Essex, and Hudson) with highest rates of cervical cancer incidence (9.10–11.40 per 100,000) and mortality (2.20–3.80 per 100,000).

## Discussion

In this study, we estimated utilization rates of HR-HPV DNA testing and examined the factors associated with HR-HPV DNA testing and HR-HPV infection among a diverse sample of medically underserved women in New Jersey seeking cervical cancer screening services through NJCEED. We observed relatively low rates of HR-HPV test utilization (26.6%) among this sample of eligible women. We also observed wide variation in HR-HPV DNA test utilization and HR-HPV infection by individual-level (age, race/ethnicity, birthplace, cervical cancer screening patterns) and area-level characteristics (poverty), as well as by county of residence. Of note, while non-Hispanic Black and Hispanic patients had higher odds of HR-HPV DNA test use, they were significantly less likely to be HR-HPV-infected compared to NHWs.

Few studies have examined the rates of HR-HPV DNA testing in cervical cancer prevention [9–12], but they yielded similarly low utilization as we report here. Cuzick et al. [11], in the first US study to report on population-based utilization of HPV DNA testing (specifically co-testing) in cervical cancer screening, showed HPV test utilization was 11.9% among women 15–65 years from 2007 to 2012 in New Mexico. Similar to our findings of an increase in the rates of HPV testing across the study period, the study by Cuzick and colleagues also showed an increase in HPV co-test utilization among women 30–65 years from 5.2% in 2007 to 19.1% in 2012 [11]. Another study [10], which estimated HPV testing in cervical cancer using laboratory and administrative data from Johns Hopkins Hospital found that between February 2004 and December 2007, HPV co-test utilization among women ≥30 years was 7.8% in Baltimore, Maryland. This study also showed an increase in test utilization from 2.2% in 2005 to 15% in 2006, which plateaued around 13% in 2007 [10]. A study examining data from 4 health systems (Group Health Cooperative [Washington], Kaiser Permanente Northwest [Oregon/Washington], Kaiser Permanente Hawaii, and Reliant Medical Group [Massachusetts]) between 1998 and 2007 [12], found that HPV co-testing became the guideline for these health systems in 2005 and that the rate of HPV co-testing was 130 per 1000 person-years in 2007.

Similar to our current study, another study [9] examining HPV DNA test utilization (although not focusing on co-testing explicitly) between July 2001 and June 2006 among Florida Medicaid enrollees found that only 2.9% of Medicaid beneficiaries received a HPV DNA test during the study period. This study showed that HPV DNA test use increased over the study period from 0.6% in 2001 to 9% in 2006 [9]. Our study showed that 26.6% of women who sought cervical cancer screening through NJCEED from 2009 to 2015 were tested at least once for HR-HPV and among them, 13.3% tested positive for HR-HPV infection. Almost all of the women who tested positive in this study (99.4%) had their first HR-HPV DNA test as a co-test with Pap test. Findings from our study as well as these previous studies [9–12] highlight the low utilization of HPV DNA testing among uninsured/underinsured, vulnerable populations in several geographic regions in the US, which may be due to lack of providers’ recommendations for HPV testing for various reasons (e.g., high costs/limited funding, providers’ practices and beliefs, etc.). While emerging data, based on hospital pathology records [13] and healthcare claims data [14] suggest increasing uptake of HPV co-testing, little data exist on utilization among hard to reach, uninsured and underinsured populations. Additional studies are needed to understand the barriers to utilization of HR-HPV DNA testing, particularly among high risk populations, and to develop strategies to increase the utilization rates of this important cervical cancer screening test.

We observed wide variation in HR-HPV DNA test utilization and HR-HPV infection by individual-level and area-level characteristics. After controlling for covariates, NHBs, APIs, and Hispanics (compared to NHWs) and residents of ZCTAs with larger proportions of residents living below FPL (quintile 5 vs. quintile 1) had higher odds of ever being tested for HR-HPV. The higher odds of HR-HPV DNA testing among NHBs, Hispanics, and women living in high poverty areas were particularly interesting given that there are striking disparities in the cervical cancer burden in these groups, despite widespread reductions in incidence and mortality. Minority, uninsured and low socioeconomic status (SES) women tend to be screened less often and this lower screening tends to result in increased cervical cancer morbidity and mortality [19–21]. These findings suggests that NJCEED is generally reaching the appropriate target population in these areas (minority and low SES women) for cervical cancer screening, which will ultimately have an impact on cervical cancer burden in the state of New Jersey and indicate that although the women seeking screening through NJCEED are medically underserved, they may very well be more health-seeking than expected (in terms of cervical cancer screening). Prior studies have shown reduced barriers to accessing care (e.g., transportation, walkability, availability of clinics) and in some cases increased health care utilization among individuals residing in the most inner city, socioeconomically deprived areas compared to those residing in less socioeconomically deprived areas outside of the immediate inner city [22–26]. Furthermore, geographic variations in testing across the state and lower screening rates in specific NJCEED Program areas, particularly more rural areas with low SES women, warrants a focus on targeted strategies for women living in these areas who may face different barriers than urban, minority women.

We also found that the odds of HPV testing decreases as age increases, a finding which has been reported by other investigators [10]. Additionally, we found that women born in Central and South America and the Caribbean had lower odds of ever having a HPV DNA test compared to US-born women. While no other study has reported this association, lower Pap test utilization has been previously reported among foreign-born women [27–31]. Given the projected changes to the composition of the US population, in which the population of native and foreign-born minorities is expected to grow substantially [32], it is essential that cervical cancer prevention and control initiatives begin to focus on developing effective strategies for increasing cervical cancer screening, particularly among foreign-born women, as they tend to experience greater barriers to cancer screening [27–30] and may be at increased risk of cervical cancer (compared to US-born women) due to the prevalence of HPV infection and other risk factors in their native countries [33, 34].

We found that among women who ever had a HPV DNA test, 13.3% of them had at least one positive test, indicating infection with one or more HR-HPV types. Our finding that NHB and Hispanics had lower odds of ever having a positive HPV test, although having higher odds of ever having a HPV DNA test, was unexpected, given existing data that show higher rates of HPV infection among low SES, minority women, which has been linked to their disproportionate burden of cervical cancer [19, 21, 35–38]. It is unclear whether women reached by NJCEED are inherently more activated individuals and therefore may have other characteristics that relate to decreased prevalence of HR-HPV infection. Of particular importance would be further examination of the risk factors associated with HR-HPV type-specific infection among women screened for cervical cancer through NJCEED, which would have implications related to the benefit of HPV vaccination for cervical cancer prevention among some subgroups. Furthermore, such data will also contribute to the development of targeted interventions to promote improved cervical cancer control among minority and medically underserved women.

The observed variation in HR-HPV DNA test utilization by New Jersey county of residence identified several counties with rates higher than the average of the study sample, which indicates that NJCEED providers covering these areas are doing relatively well at providing guideline cervical cancer screening in medically underserved populations in the state. However, we also identified several counties that had HPV screening rates that were quite low. While we did not examine provider characteristics in the current study, we hypothesize that they are important to understanding variation in cervical cancer screening and HPV DNA testing across counties and by NJCEED providers. Prior studies have shown that variation exists in providers’ adherence to screening guidelines and specifically in recommendations for HPV testing [16, 39–42].

The strengths of this study include its large sample size of diverse, uninsured and underinsured women who sought cervical cancer screening services through NJCEED during the study period. Availability of cervical cancer screening history through large administrative data for several years was also a strength. An additional strength is that our findings will contribute to the dearth of population-based data related to HR-HPV test utilization and HR-HPV infection in a diverse sample of women. There were also some limitations of this study that should be considered in the interpretation of our findings. First, the use of administrative data from the NJCEED Program limited the availability of additional covariates of interest, including specific individual-level SES measures and more detailed information on sexual behaviors and other risk factors for HR-HPV infection and HPV vaccination status among study participants. Additionally, we cannot confirm that women included in this study did not seek cervical cancer screening outside of NJCEED during the study period. We also saw a high proportion of women who were only screened once within the program, based on their encrypted participant ID number. While some of these women may have repeat screenings within NJCEED across our study years, they may have been assigned a different participant ID due to screening at a different location or other reasons. Another limitation was that we don’t know if any of the NJCEED providers or sites targeted screening for high-risk groups (and the variation among providers), which could explain some of the observed differences in county-level testing rates. We also don’t know if NJCEED is reaching all of the women who are eligible for the program and if all of these women are seeking cervical cancer screening services through the program, if providers are meeting their catchment area targets for the NJCEED Program, and how much funding they have to meet the needs of the women in their catchment area. Thus, generalizability of these data to women not eligible for NJCEED services is not possible. And finally, lack of data on specific HR-HPV genotypes prevented us from examining the type specific prevalence of HR-HPV by subgroup of women, which would really enhance our assessment of cervical cancer risk in this population.

## Conclusions

This is one of the few studies to examine utilization of HR-HPV DNA testing and HR-HPV DNA infection among a sample of medically underserved women who are at high-risk for cervical cancer. These study findings provide insight to individual, area-level factors associated with testing and positive testing results as well as geographic variation across the state in HR-HPV DNA testing, which can inform further exploration of barriers to low utilization of HR-HPV DNA testing, particularly among high risk populations and to develop strategies to increase the utilization rates of this important cervical cancer screening test.

## Abbreviations

API: Asian/Pacific Islander
CaST: Cancer Screening and Tracking System
CDC: Centers for Disease Control and Prevention
DNA: Deoxyribonucleic acid
FDA: Food and Drug Administration
FPL: Federal poverty level
HR-HPV: High-risk human papillomavirus
MDE: Minimum Data Element
NBCCEDP: National Breast and Cervical Cancer Early Detection Program
NCI: National Cancer Institute
NHB: Non-Hispanic Black
NHW: Non-Hispanic White
NJBCCCI: New Jersey Breast and Cervical Cancer Control Initiative
NJCEED: New Jersey Cancer Early Education and Detection
NJDOH: New Jersey Department of Health
NJSCR: New Jersey State Cancer Registry
SEER: Surveillance, Epidemiology, and End Results Program
US: United States
ZCTA: Zip code tabulation area
